# The Relationship between Self-Esteem, Self-Efficacy, and Career Decision-Making Difficulties: Psychological Flourishing as a Mediator

**DOI:** 10.3390/ejihpe13090113

**Published:** 2023-08-23

**Authors:** Anne Pignault, Merlin Rastoder, Claude Houssemand

**Affiliations:** 1 2LPN (Psychology and Neuroscience Lab, UR7489), Université de Lorraine, 54000 Nancy, France; 2 Institute for Lifelong Learning and Guidance, Department of Education and Social Work, University of Luxembourg, L-4366 Esch-sur-Alzette, Luxembourgclaude.houssemand@uni.lu (C.H.)

**Keywords:** career decision-making difficulties, self-esteem, self-efficacy, flourishing, positive psychology, structural equation modeling

## Abstract

Well-being associated with optimism, social support, and self-esteem is positively correlated with career decision. In this perspective, a rather recent concept of *flourishing* as an integrative notion incorporating these different resources, positive affect, and positive experience is particularly relevant to better understand the relations and correlations between self-evaluation, well-being, and career decision-making difficulties. The present study then examines the relationship between these possible difficulties, self-esteem, self-efficacy, and flourishing. One hundred and seventy-two higher education students participated in the study and completed a four-part questionnaire with the *Career Decision-Making Difficulties Questionnaire,* the *Flourishing Scale*, the *Rosenberg Self-esteem Scale*, and the *General Self-Efficacy Scale*. The results highlight the mediation of psychological flourishing between personality dimensions—self-esteem and self-efficacy—and career decision difficulties and the fact that those who are most flourishing in their lives will also have the least difficulty in making a career decision. As for the practical implications, guidance counselors for students and young adults need to identify resources and difficulties they have to cope with. This study emphasizes the importance of guiding students in three areas: self-esteem, the perceived quality of social relations, and the meaning attributed to one’s existence. Finally, the contribution of positive psychology to career development will be discussed.

## 1. Introduction

While many decisions are easy to make most of the time, others are more complicated and even stressful [1]. Since 1996, Gati et al. [2] have pointed out that choosing a career is among the most complex and important decisions that individuals face in life.

In our modern society where the world of work is constantly changing, from changes in educational systems or increased specialization in training to difficulties in finding a job or managing increasing transitions, making decisions about education, occupation, and career is indeed particularly challenging.

Thus, many studies have first investigated career indecisiveness [3], career indecision process and profiles [4], and career decision-making difficulties [2,5,6] and then examined the relationship between career decision-making difficulties and many other individual or psychological variables [7,8,9]. Some of these studies have notably pointed out a correlation between well-being and career (in)decision [10,11].

In line with this research and in order to further understand the difficulties in career decision-making, the present study attempts to highlight the existing relationship between these difficulties, self-esteem, self-efficacy, and flourishing—a very relevant integrative concept linked to the positive psychology movement, which makes it possible to consider the contribution of positive psychology in career development and career decision.

### 1.1. Understanding Career Decision-Making Difficulties

Career (in)decision is a rather complex concept and researchers have adopted several different approaches over time to study this phenomenon. Forner [12] distinguishes four of them: a dichotomous approach, a developmental approach, a cognitive approach, and a multidimensional approach.

Since the 1970s, many articles have sought to distinguish between indecision and indecisiveness [13]. While indecision refers to the inability to make a career decision [2] that nevertheless seems to correspond to a normal, temporary developmental phase, indecisiveness refers to a personality trait [5,7,9,14].

Thus, to better understand the various problems potentially related to career indecision and to be able to propose relevant counseling actions, the authors highlighted the need to specify the concept and to distinguish various categories of difficulties. In 1996, Gati et al. [2] developed the Career Decision-Making Difficulties Questionnaire (CDDQ). Its structure (very recently confirmed again across 13 countries by Levin et al. [15]) consists of 10 subscales, or 10 difficulties categories, divided into three groups: *Lack of Readiness* due to lack of motivation, indecisiveness, dysfunctional myths, and lack of knowledge about the process; *Lack of Information* about self, occupation, and ways of obtaining information; *Inconsistent Information* involving unreliable information, internal conflicts, and external conflicts [2] (p. 512). Establishing the reasons for career indecision is indeed one of the first steps in career counseling, the CDDQ, adapted to different countries and languages, is an indispensable tool widely used for this purpose [15].

### 1.2. Linking Career Decision-Making Difficulties, Self-Esteem, and Self-Efficacy

In a recent meta-analysis of 86 studies, Udayar et al. [16] highlighted the association between difficulties in career decision-making and self-evaluations. Self-evaluations, “which refer to individuals’ global and situational evaluations about themselves and their abilities” [16] (p. 611), have been essentially understood with two main constructs, self-efficacy and self-esteem, and many studies have demonstrated close links between these two constructs and career decision-making.

The role of self-efficacy as a fundamental influence on career indecision has been highlighted by empirical research in recent decades [16,17,18]. Bandura’s social cognitive theory [19] is one of the key theories for understanding feelings of efficacy. This theory aims to identify the role of self-efficacy beliefs, better known as a sense of personal efficacy, in the regulation of action. Self-efficacy beliefs refer to a person’s beliefs about their ability to motivate themselves, activate their cognitive resources, and plan the behaviors necessary to exert control over the events in their lives. It implies the ability an individual believes he or she possesses to achieve a given goal, which in the case of this study is career decision-making. An individual’s effective functioning thus requires not only skills but also efficacy beliefs that will enable him or her to use them effectively [20]. In terms of career decision-making, Taylor and Betz [21] have talked about the “sense of vocational efficacy”—a concept derived from the sense of self-efficacy, referring to a person’s beliefs about his or her ability to perform a task requiring career decision-making. It is worth mentioning that the social cognitive theory of educational and vocational guidance [22] is one of the theories that help to explain the link between the concept of a sense of efficacy and that of vocational indecision. This theory suggests that the relationship between personal characteristics (such as personality) and career outcomes such as indecision is mediated by self-efficacy. Self-efficacy, then, is a predictor of career indecision or decisiveness [22,23,24,25]. Guay and colleagues [26] have come to the same conclusion in a longitudinal study. Indeed, these researchers have found that the decrease in indecision is induced by an increase in the level of a feeling of efficacy in relation to work activities. In his work, Rossier [27] has proposed considering self-efficacy a regulatory process in the “expression of career-related behaviors” (p. 161). Later, in a paper presenting the validation of the French version of the CDDQ, Rossier et al. [8] showed that self-efficacy moderates the negative link between self-esteem and career decision difficulties.

According to Rosenberg [28], self-esteem is an attitude toward oneself that can be positive or negative. Orth et al. [29] define it as “a person’s subjective evaluation of his or her worth as a person” (p. 1046). Thus, high self-esteem means that an individual values himself or herself, respecting himself or herself for what he or she is. This indicates the extent to which the person considers him or herself important, worthy, capable, and able to succeed. In this regard, self-esteem has a double connotation, affective and cognitive [30]. Many studies point out that self-esteem plays an important role in the development of career maturity and the subsequent decision-making process or even future career characteristics [31]. More precisely, the literature highlights a relationship between low self-esteem and career indecision [8,9,32,33]. Moreover, Choi and colleagues’ [18] meta-analysis shows that self-esteem is positively correlated with self-efficacy in career-decision making. Betz et al. [23] have also noted that self-esteem influences a student’s career decision-making self-efficacy. Lent and Fouad [34] have argued that although self-efficacy and self-esteem are similar in many ways, they are two distinct concepts. As such, people with high self-esteem tend to have positive feelings about themselves, while those with low self-esteem tend to have negative feelings about themselves and these feelings persist even when they believe they are highly competent. Lent and Fouad [34] have observed that individuals with high self-esteem engage in actions oriented at proficiency, thus resulting in higher job productivity, higher career decision-making self-efficacy, life satisfaction, and better psychological outcomes.

A review of contemporary studies demonstrates a lack of consensus on the type of correlation between self-efficacy and self-esteem. In particular, Bandura [19] has noted that having efficacy expectations related to executing or accomplishing some job could impact one’s views on self-esteem, especially if success or failure of that job is strongly connected to his or her self-worth. On the other hand, research by Lane et al. [35] and Dogson and Wood [36] has shown that self-esteem influences changes in self-efficacy. Finally, in an educational context, Lane et al. [35] “believe that self-esteem is likely to flow from perceived efficacy expectations, rather than the reverse” (p. 254). Regarding career decisions, it has been established that the pursuit of higher education and training is tied to an individual’s self-worth.

Rossier et al. [8] have also indicated that a higher level of self-esteem is negatively correlated with career indecision and positively correlated with career exploration, which also means that one is more career decisive. The results obtained in their study further clarify the relationship between the three concepts by showing that “self-efficacy partially mediated the relationship between self-esteem and career decision-making difficulties” (p. 10).

### 1.3. Examining the Relationship between Career Decision-Making Difficulties, Self-Esteem, Self-Efficacy, and Well-Being

The literature makes it possible to establish single or multiple correlations between the constructs of career decision-making difficulties, self-esteem, self-efficacy, and well-being.

In 2004, Mann [37] explored the link between self-efficacy, self-esteem, and mental health outcomes. The scholar found that self-efficacy is positively correlated with self-esteem and concurred with Bandura [19] who affirmed that “the levels of self-esteem and self-confidence can influence self-efficacy” (p. 365) and showed a direct link between self-esteem, happiness, and mental wellbeing.

Reardon et al. [38] applied the theory of cognitive information processing (CIP) to investigate the influence of self-concept on career selection and students’ mental wellbeing. The authors [38,39] noted that effective career decision-making is based on the processing of information pertaining to self-awareness, occupational knowledge, and decision-making strategies. The scholars expressed an opinion that self-knowledge is the most important factor in decision-making as it relates to how a student perceives his or her interests, values, and competencies. Reardon et al. [38] concluded that low self-confidence and self-efficacy are connected to negative career thoughts, which in turn cause commitment anxiety, adverse impacts on vocational development, and deterioration of an individual’s wellbeing. Saunders et al. [40] added that negative career thoughts inhibit the career decision-making process and increase career indecision. Bullock-Yowell et al.’s study [41] found that youths with high career decision-making self-efficacy have a low likelihood of negative career thoughts and were well adjusted in their career journey. According to the study, people with high self-esteem and high self-efficacy tend to map out a clear path for their success and seek help to achieve career goals. In contrast, low self-esteem and low self-efficacy are the strongest predictors of career indecision and poor psychological well-being outcomes.

Isik [42] established that positive affect is more influential on career decision self-efficacy than negative affect. These findings concur with previous studies by Hammond et al. [43] and Betz et al. [44] which established that negative affect is not a reliable predictor of career decision self-efficacy and that positive affect is strongly correlated with career decisiveness and optimism. These studies concluded that an individual’s self-esteem and self-efficacy influence their affective states and psychological health. Isik [42] further concurred that career guidance interventions significantly improve students’ mental health by reducing their anxiety levels, increasing their knowledge in a preferred area of study, their confidence in their self-concept, and their career decision self-efficacy.

In 2004, Creed et al. [45] established a positive link between well-being and career adaptation and indicators of career decisiveness. Furthermore, the scholars indicated that high self-efficacy, optimistic attitudes, and self-esteem are strong predictors of career decisiveness. The authors asserted that career decisiveness could take place due to lower levels of pessimism and a reduced likelihood to perceive barriers along the academic journey. Later, in 2018, Creed et al. [46] revealed that vocational identity was connected to goal setting, goal management, and self-efficacy. In particular, Creed et al. [46] examined self-efficacy in relation to how youths evaluate available resources and how they believe they could maximize them to select their preferred career from a list of career options.

In turn, Walker and Peterson [11] investigated the relationship between career thoughts, indecision, and the symptoms of depression. The researchers concluded that career decision and mental health issues are often intertwined; however, they did not define the nature of this relationship and they explained that the influence could flow in both directions, with mental health affecting career indecision and career indecision affecting mental health.

Finally, a review of recent studies by Redekopp and Huston [47] further demonstrated that career development is strongly connected to mental health. According to these authors, mental health has a reciprocal relationship with career development, work, and wellbeing. It is critical to note that mental health is different from mental illness, even though many stakeholders consider them two ends of a continuum. Keye’s model [48] indicates that mental health and mental illness have an inverse relationship whereby low or no mental illness translates to high mental health, and high mental illness translates to low mental health. Therefore, Keye’s theory [48] has been used to interpret literature on the impacts of career choices on mental health.

Positive affect, health, and well-being are thus essential conditions for career decision-making [9,11,41,46,49]. More precisely, the literature has highlighted the positive impact of several resources, such as optimism, social support, and self-esteem, fostering career decision-making. In order to explore this further, it seems worth questioning and considering the contributions of positive psychology to career development and career decision.

### 1.4. Considering the Contribution of Positive Psychology

*Positive psychology* can be defined as “the study of the conditions and processes that contribute to the flourishing or optimal functioning of people, groups, and institutions” [50] (p. 104). In recent years, despite the criticism directed toward this movement [51], several authors have highlighted the value of developing positive experiences and affects in different areas of life, including career development, education, and guidance [52]. Some studies have also examined more specifically the links between positive affect and success or achievement, ultimately demonstrating a bidirectional relationship —for example, [53,54], a virtuous circle [52] in which positive affect and mood lead to positive experiences, which in their turn lead to further positive affect. It therefore seems important to take these results into account to better understand the constraints and strengths for career decisions and to examine in greater detail the significance of the links between positive affect and possible difficulties in making career decisions.

Thus, the rather recent concept of flourishing [55,56,57] as an integrative notion incorporating different components mentioned above is particularly relevant to better understand the relations and correlations between self-evaluation, well-being, and career decision-making. In 2002, Keyes [55] introduced the concept of flourishing, describing the presence of mental health and operationalizing mental health as “a syndrome of symptoms of positive feelings and positive functioning in life” (p. 208). The Flourishing Scale developed by Diener et al. [56] measures the main aspects of psychological and social-psychological functioning “from positive relationships, to feelings of competence, to having meaning and purpose in life” (p. 146). Later, consistent with previous studies, Ramirez-Maestre et al. [58] determined that flourishing seems to be a process that could explain the association between psychological factors such as anxiety, optimism, pessimism, and positive affect. In our area, it appears again that psychological flourishing is an illuminating concept for a better understanding of career decision-making difficulties.

### 1.5. The Present Study

Thus, to further understand career decision-making difficulties, we intend to examine the relationships between these possible difficulties, flourishing, self-esteem, and self-efficacy. More precisely, in line with previous studies and results, we first hypothesize negative correlations between career decision difficulties (general score or the three subscores—lack of readiness, lack of information, and difficulties related to inconsistent information) and self-esteem and self-efficacy (H1).

Then, based on recent research proposing a mediation model of the impact of certain personality characteristics on career decision difficulties and pointing out the links between career decision-making difficulties, self-esteem, self-efficacy, and well-being, we hypothesize that the relationship between self-esteem and self-efficacy and the three career decision difficulties is mediated by flourishing (H2).

## 2. Materials and Methods

### 2.1. Participants

The sample for this study consisted of 172 students (111 females [64.5%] and 61 males [35.5%], Mage = 23.88 years, SDage = 4.29, age range: 18 to 41 years). The gender imbalance is explained mainly by the predominant share of female students in the study programs concerned (education, psychology, or humanities, for instance). The respondents were of Luxembourg (144) or French (28) nationality. Out of 172 students, 40 of them are pursuing a Higher Technician Certificate (technical tertiary education), 79 are pursuing a bachelor’s degree, 51 are enrolled in a master’s program, and 2 are in a Ph.D. program at the University of Luxembourg. In addition, 46% (*n* = 79) of the students would like to work after graduation, 36% (*n* = 62) would prefer to continue their studies, and 18% (*n* = 31) do not yet know what to do after graduation. It should be noted that the sample of respondents contains only students but some of them are in an upskilling or reskilling phase. This explains why some participants in the study are older than others and beyond the typical age of higher education students.

### 2.2. Measures

The *Career Decision-Making Difficulties Questionnaire* (CDDQ). The validated version of the CDDQ [8] is composed of 34 items grouped into 10 difficulty subfactors that describe either one general factor of career decision-making difficulties or three second-order factors (see the description above, in the theoretical part). The items are rated on a 9-point Likert-type scale ranging from 1 (does not describe me) to 9 (describes me well), and two of them are control items usually not included in the computed scores. The scale has been used extensively in different studies and has adequate psychometric qualities. For example, Gati and Saka [59] showed internal consistency values of alpha = 0.91 for the full scale and the following values for each of the sub-dimensions respectively: alpha = 0.62 (lack of readiness), alpha = 0.88 (lack of information), and alpha = 0.87 (inconsistent information). The French version of the scale used in the present study has been psychometrically validated by different research [60,61]. Recently Rossier et al. [8] showed the adequacy of the factorial structure of the French version of this scale on several French-speaking samples and estimated its internal consistency at 0.93 for the entire score and at 0.57–0.93 for the three subfactors. Item examples for each main dimension (in the order described above) are: “I believe that I do not have to choose a career now because time will lead me to the ‘right’ career choice”; “I find it difficult to make a career decision because I do not know what steps I have to take”; and “I find it difficult to make a career decision because I have contradictory data about the existence or the characteristics of a particular occupation or training program.” In our analyses, we present the two confirmatory factor analyses corresponding to the two possible measures with this scale (one or three factors [59]). Then, we use the three-factor solution in the structural equation modeling to make our results comparable with previous validation studies of the French scale, particularly that of Rossier et al. [8].

The *Flourishing Scale* [62]. It individually measures specific concepts of psychological well-being, such as universal human psychological needs, meaning and purpose in life, engagement in activities, optimism, positive social relationships, contribution to the well-being of others, self-acceptance, and respect of others for oneself. The scale, composed of 8 items ranging from 1 (strongly disagree) to 7 (strongly agree), demonstrates very good psychometric qualities with a consistency of 0.87, temporal stability of 0.71, and inter-item correlations between 0.57 and 0.71. Higher scores represent higher levels of psychological well-being, resources, and strengths. The French version of this scale was used in the present study. It has good psychometric properties, and factor analyses have confirmed its unidimensional factor structure [63]. An item example is: “I lead a purposeful and meaningful life”.

The *Rosenberg Self-Esteem Scale* [28]. This scale evaluates the respondent’s feelings of self-worth through 10 statements rated from 1 (strongly disagree) to 4 (strongly agree). Higher scores represent a higher level of self-esteem. This scale is one of the most widely used ones to measure this psychological construct, and it has an internal consistency of alpha = 0.88. The French version was used in the present study [64]. It has an internal consistency between alpha = 0.83 and 0.90 and a test-retest stability index of 0.84. An item example is: “I think I have a number of good qualities”.

*General Self-Efficacy Scale* [65]. The construct of perceived self-efficacy reflects an optimistic self-belief [66]. Unlike other scales designed to assess optimism, this one refers explicitly to personal agency, i.e., the belief that one’s actions are responsible for one’s successes. Perceived self-efficacy is a forward-looking and operational construct. The scale is composed of 10 items referring to successful coping and implying an internal-stable attribution of success—for example, “I can solve most problems if I invest the necessary effort”. Each item is coded from 1 (not at all true) to 4 (exactly true). In samples from 23 nations, Cronbach’s alphas range from 0.76 to 0.90, with the majority in the 0.80. The French version of this scale has been validated and used in several Canadian studies [67]. We chose to use general self-evaluation—self-esteem and self-efficacy [16,68]—since these two variables are considered a stable trait [30].

### 2.3. Procedure

The study was proposed via social networks to students from all fields of study at the University of Luxembourg and high schools offering higher academic education. The self-assessment questionnaires were administered via the LimeSurvey platform. Respondents were informed of the purpose of the survey, that their anonymity would be respected, and that they could withdraw from the survey at any time without any consequences for them. Additionally, the Luxembourg Agency for Research Integrity specified that according to Code de la santé publique, Article L1123-7, research does not require Research Ethics Committee (Les Comités de Protection des Personnes) approval if it is non- biomedical, non-interventional, and observational, and if does not collect personal health information. Therefore, CNR approval was not required. Only complete surveys were used for the analyses. 

The statistical analyses were carried out using the R packages: Psych for descriptive, correlational, and inferential statistics; and Lavaan for structural equations modeling (SEM), including confirmatory factors analyses (CFA) and path analysis. All SEMs were carried out based on the responses to the different items in the questionnaires. In order to respect the level of measurement of the proposed constructs in the form of Likert-type scales, the analyses were based on polychoric correlation matrices [69] using the SEM diagonally weighted least squares (DWLS) estimator [70]. None of the SEM analyses were based on composite scores, and the differential structural analyses of direct and indirect effects were performed using the bootstrapping method.

According to the literature [71,72], the *χ^2^*/*df* (<3), *CFI* (>0.90 or >0.95), *TLI* (>0.90 or >0.95), and *RMSEA* (<0.08 or <0.05) were used to judge the quality of the retained models.

Regarding the sample size of the study, based on recent methods for determining the minimum number of subjects needed to test SEMs [73,74] and on the results of recent studies using the same scales [8], Soper’s sample size estimation platform [75] determined that our model composed of 16 latent variables and 60 observed variables required a number of subjects between 116 and 170 (between 141 and 168 for the model with 15 latent variables and 52 observed variables). The sample size of the present study is therefore in line with requirements to ensure statistical power level of the results presented [76].

Although our sample size meets the statistical requirements for this type of analysis, we felt it was important to confirm our results by using another SEM method not based on covariance (CB-SEM). This statistical method is experiencing a surge of interest and is called PLS-SEM (partial least squares structural equations modeling). Although the two methods have the same aim of empirically verifying theoretical models, the former is more oriented toward an exploratory modeling approach (seeking an explanatory model for the empirical data by verifying causal links between latent factors) [77], while the latter is considered to be more of a “causal-predictive” method (in particular by allowing the results to be generalized outside the sample used for modeling) [78]. Hair et al. (2017) believe that the use of this method is important, particularly when the sample size is small [79]. The cSEM package of R was used to carry out these complementary analyses.

## 3. Results

### 3.1. Confirmatory Analyses

First, confirmatory factor analyses were carried out for each of the four scales in the study to check their structure. The results are presented in Table 1.

In general, all scales in the study have structural indices that are in line with expectations and standards.

### 3.2. Self-Esteem, Self-Efficacy, and Career Decision-Making Difficulties

In their article, Rossier et al. [8] highlighted negative correlations between the general career decision difficulties score or the three subscores of this scale (lack of readiness, lack of information, and difficulties related to inconsistent information) and self-esteem and self-efficacy. Correlational results similar to those reported by these authors for their Swiss sample are found in the present study (see Table 2) and confirm its H1 hypothesis. Only the correlation between the scores of self-esteem and self-efficacy is higher than in the previous study. This can certainly be explained by the fact that the scale used here measures general self-efficacy and not career-specific self-efficacy.

**Figure 1:**
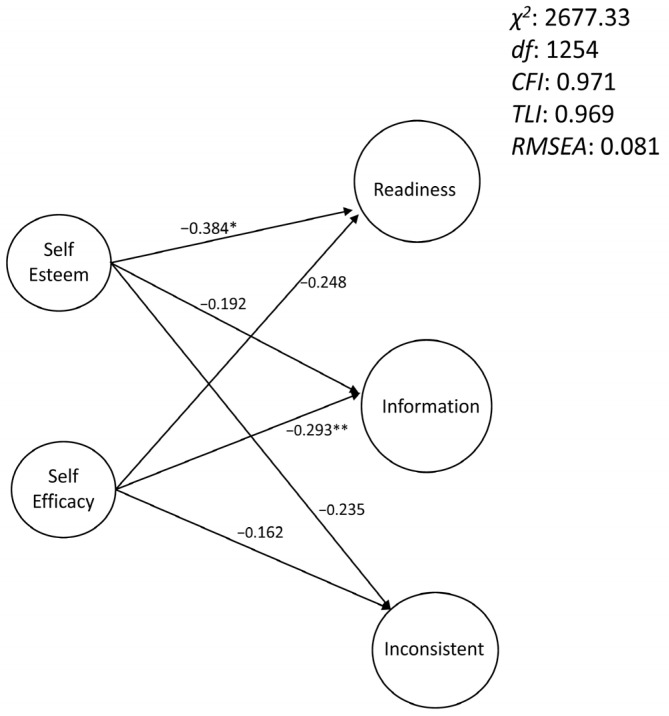
Path analysis of self-esteem, self-efficacy, and career decision difficulties. χ2: chi-squared; df: degrees of freedom; CFI: comparative fit index; TLI: the Tucker-Lewis index; RMSEA: root mean square error of approximation; Readiness: lack of readiness; Information: lack of information; Inconsistent: difficulties related to inconsistent information; *: *p* < 0.05; **: *p* < 0.01. Each latent variable was defined by all the items of the corresponding scale (to simplify, no items were represented in the figure).

The mediation of self-esteem through self-efficacy, proposed by Rossier et al. [8], was analyzed using a structural equation model and the bootstrapping method. The results are not in line with expectations; self-esteem has no direct effect on the three dimensions of the CDDQ (lack of readiness: 95% CI = [−0.784, 0.020]; lack of information: 95% CI = [−0.558, 0.066]; difficulties related to inconsistent information: 95% CI = [−0.541, 0.018]). The indirect links through mediation of self-efficacy are also not significant (lack of readiness: 95% CI = [−0.592, 0.158]; lack of information: 95% CI = [−0.560, 0.014]; difficulties related to inconsistent information: 95% CI = [−0.416, 0.117]). In contrast, a simpler unmediated model shows direct effects of self-esteem on the dimension of lack of readiness and direct effects of self-efficacy on the dimension of lack of information. Figure 1 illustrates these results.

### 3.3. Self-Esteem, Self-Efficacy, Career Decision-Making Difficulties, and Flourishing

In order to verify the links between career decision difficulties, personal flourishing, self-esteem, and self-efficacy, a SEM model was developed. This model is based on the results highlighted above, with the addition of a mediation of flourishing. The indicators of model fit are very good ensuring that the empirical data confirm the theoretically assumed links between the latent variables.

**Figure 2:**
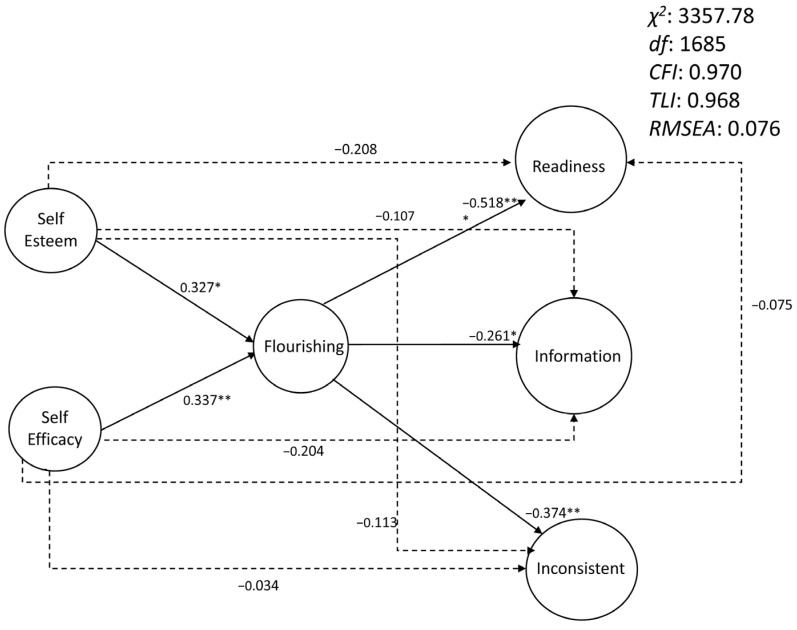
Path analysis of the self-esteem, self-efficacy, flourishing, and career decisions difficulties (direct and indirect effects). χ2: chi-squared; df: degrees of freedom; CFI: comparative fit index; TLI: the Tucker-Lewis index; RMSEA: root mean square error of approximation; Readiness: lack of readiness; Information: lack of information; Inconsistent: difficulties related to inconsistent information; *: *p* < 0.05; **: *p* < 0.01; ***: *p* < 0.001. Each latent variable was defined by all the items of the corresponding scale (to simplify, no items were represented in the figure).

The direct links between the three factors of career decision difficulties and self-esteem (lack of readiness: 95% CI = [−0.592, 0.174]; lack of information: 95% CI = [−0.399, 0.161]; difficulties related to inconsistent information: 95% CI = [−0.368, 0.225]) and self-efficacy (lack of readiness: 95% CI = [−0.470, 0.280]; lack of information: 95% CI = [−0.542, 0.077]; difficulties related to inconsistent information: 95% CI = [−0.328, 0.224]) are not significant as evidenced by a bootstrapping analysis on the SEM model. In contrast, the indirect effects between self-esteem and self-efficacy and the three career decision difficulties mediated by flourishing are significant. The links between self-esteem, self-efficacy, and flourishing are positive (respectively, 95% CI = [0.005, 0.648]; 95% CI = [0.080, 0.635]), and those between flourishing and decision difficulties are negative (lack of readiness: 95% CI = [−0.653, −0.164]; lack of information: 95% CI = [−0.513, −0.067]; difficulties related to Inconsistent Information: 95% CI = [−0.546, −0.96]). These results confirm the H2 hypothesis of the present study. Figure 2 shows the whole model.

**Figure 3:**
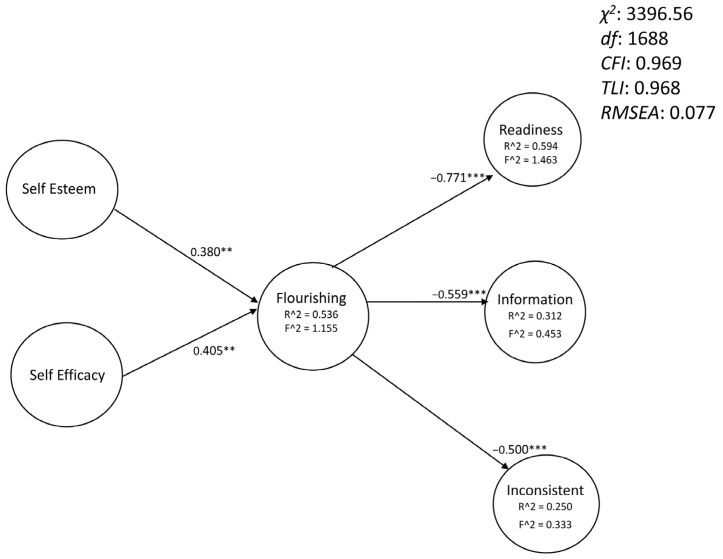
Path analysis of the self-esteem, self-efficacy, and career decisions difficulties with mediation of flourishing. χ2: chi-squared; df: degrees of freedom; CFI: comparative fit index; TLI: the Tucker-Lewis index; RMSEA: root mean square error of approximation; Readiness: lack of readiness; Information: lack of information; Inconsistent: difficulties related to inconsistent information; **: *p* < 0.01; ***: *p* < 0.001. Each latent variable was defined by all the items of the corresponding scale (to simplify, no items were represented in the figure).

As the direct effects of self-esteem and self-efficacy on career decision difficulties were not significant, a final SEM model including only the effects mediated by flourishing was finally proposed. It is presented in Figure 3.

Using a PLS-SEM analysis, the results are confirmed with lower levels of variance explained for the variables in the model. First, the variance inflation factor (VIF), which measures the amount of multicollinearity in regression analysis, was determined for each part of the path analysis. It turns out that the vast majority of these are between 1 and 2 (indicating low multicollinearity between the independent variables of the regressions performed), and all do not exceed 5 (a value indicating average multicollinearity). As a result, the relationships between the latent variables in the model are not altered by the correlations between the indicators that determine them (independent variables). With this analysis, the R^2^ values are as follows: Flourishing (0.377; f^2^:0.605), Readiness (0.274; f^2^:0.377), Information (0.190; f^2^:0.235), and Inconsistent (0.178; f^2^:0.216), indicating substantial to moderate effects [76].

## 4. Discussion

The structural analysis of the CDDQ on a sample of Luxembourg students confirms the results recently highlighted by Rossier et al. [8] and reinforces their conclusions on the appropriateness of the Francophone version of this scale by adding a country not taken into account in their study.

In their article, Rossier et al. [8] hypothesized and verified that the effects of self-esteem on career decision difficulties are partially mediated by self-efficacy. Indeed, self-esteem could be considered a personality trait whereas subjective self-efficacy would be more contextual and dependent on expectations and adjustment [8,27]. Using another method of analysis (polychoric correlations and DWLS estimator), our results do not confirm these previous findings. Indeed, the links between self-esteem and career decision difficulties are not mediated by self-efficacy. These two personality dimensions, self-esteem and self-efficacy, have independent direct effects on two of the three dimensions of the CDDQ. Thus, self-esteem is positively related to lack of readiness, and self-efficacy is positively related to lack of information. Although the results are not identical to those previously highlighted, these two psychological constructs are strongly positively correlated with each other; therefore, the conclusions of Rossier et al. [8] remain relevant, and the work of guidance counselors is necessary to strengthen people’s self-efficacy in order to reduce the difficulties of individual decision-making in this area.

Perhaps the most important point of this research is emphasizing the mediation of psychological flourishing between personality dimensions—self-esteem and self-efficacy—and career decision difficulties. Indeed, although an inverted model in which the dependent variable would be flourishing could have been proposed, it turns out that the statistical analyses cannot validate this alternative structural proposal. The SEM analysis does not allow us to obtain a fit of the model for the data collected. The conclusion is that those who are most flourishing in their lives will also have the least difficulty in making career choices. This result also suggests that the previous recommendations should be reinforced. Guidance counselors for students and young adults need to check that people are in favorable conditions of general well-being to ensure that their decisions concerning their career choices are not biased.

If students are undecided about their career, it may be problematic for the preparation of their future and school counselors must help the students who are chronically undecided to do self and environmental exploration [80,81,82]. This broad view of indecision seems to have remained a central topic in career counseling over the past century [83]. Indeed, one of the main goals of career counseling is to facilitate the career decision-making process of counselees and, in particular, to help them overcome the difficulties they encounter during this process. In this context, it is important to understand the components of career indecision. The outcome of career exploration also possibly depends on resources that students get—for example, through social support from their families [84]. Informing the students adequately about their career perspectives will improve their career decision self-efficacy.

More specifically, the results show the importance of working on three areas: self-esteem (a result already well known), the perceived quality of social relations, and the meaning attributed to one’s existence. University students who self-evaluate to be more satisfied with life are more confident about their career choices [85]. In this way, the insights emerging from positive psychology are essential. Without resorting to positive thinking without fail, it would be interesting, based on the dimensions identified above, to propose interventions aimed at developing or reinforcing positive affect and emotion [86]. The development of positive emotions is all the more interesting because, as Fredrickson [87] explains, they are beneficial both in the short term and in the longer term, as their favorable effect continues over time. Thus, favorable impacts could be observed not only at the time of career decision but also, more generally, throughout career development. Moreover, interventions with young people and students aimed at providing more specific support for the meaning of life could be proposed [88,89]. Finally, in order to provide information on the effectiveness of these actions (a point often considered weak in the positive psychology movement), it would be interesting to carry out a follow-up.

In the future, it might also be interesting to examine the impact of current political, economic or climatic crises on young people’s personal flourishing and thus on possible career decisions difficulties. It would also be useful to consider the evolution of career models and how young people perceive and adopt them [90], to better identify any difficulties and new responses required.

Like any study, this one has its limitations. The most important one is the number and characteristics of the participants: 172 students from Luxembourg, the majority of them female. Furthermore, the labor market in Luxembourg is often perceived and described as specific [91,92], and it is possible that this economic observation also has an impact on career projections and decisions. In addition, a larger sample size could make it possible to identify and test differential variables such as gender or type of study. It will be interesting to pursue further studies in this way.

## 5. Conclusions

In recent years, many studies have sought a clearer understanding of the career decision process and career decision difficulties. Several variables were then identified as having an impact on this, in particular self-esteem, and positive links between well-being (measured on different scales) and career decision were consistently highlighted.

The present study shows that flourishing is a relevant variable for understanding what influences career decision difficulties, and it is an interesting integrative tool—avoiding multiple scales. *Flourishing* also provides an opportunity to consider the contribution of the notion of meaning in life and meaningful experience as relevant and inspiring support for interventions with students. Additionally, these results encourage to consider the contributions and recommendations of the positive psychology movement when implementing these interventions.

## Figures and Tables

**Table 1:** Confirmatory factor analyses of the scales.

Scales	χ2	df	CFI	TLI	RMSEA	Alpha	Omega
CDDQ, 1 factor	889.72	445	0.988	0.986	0.076	0.94	0.95
CDDQ, 3 factors	831.31	442	0.989	0.988	0.072	Readi: 0.70 Inf: 0.94 Inc: 0.87	0.80 0.96 0.91
FS	28.72	20	0.995	0.993	0.051	0.85	0.89
RSE	41.42	32	0.996	0.995	0.041	0.84	0.88
GSE	42.86	35	0.998	0.997	0.036	0.88	0.90

CDDQ: Career Decision-Making Difficulties Questionnaire; FS: Flourishing Scale; RSE: Rosenberg Self-Esteem Scale; GSE: General Self-Efficacy Scale; χ^2^: chi-squared; df: degrees of freedom; CFI: comparative fit index; TLI: the Tucker-Lewis index; RMSEA: root mean square error of approximation; Readi: lack of readiness; Inf: lack of information; Inc: difficulties related to inconsistent information.

**Table 2:** Correlations between the scales.

Variables	1	1.1.	1.2.	1.3.	2.
1. CDDQ					
1.1. Readi	0.82 ***				
1.2. Inf	0.93 ***	0.66 ***			
1.3. Inc	0.88 ***	0.62 ***	0.72 ***		
2. RSE	−0.38 ***	−0.34 ***	−0.37 ***	−0.28 ***	
3. GSE	−0.37 ***	−0.29 ***	−0.39 ***	−0.27 ***	0.64 ***

CDDQ: Career Decision-Making Difficulties Questionnaire; Readi: lack of readiness; Inf: lack of information; Inc: difficulties related to inconsistent information; RSE: Rosenberg Self-Esteem Scale; GSE: General Self-Efficacy Scale; ***: *p* < 0.001.

## Data Availability

The data that support the findings of this study are available from the corresponding author, A.P., upon reasonable request.
